# Mathematical Model of Emergency Indoor Air Purification by Powder Aerosol

**DOI:** 10.3390/toxics14090781

**Published:** 2026-09-03

**Authors:** Olga Kudryashova, Alexander Vorozhtsov

**Affiliations:** Faculty of Physics and Engineering, Tomsk State University, 634050 Tomsk, Russia; abv@mail.tomsknet.ru

**Keywords:** aerosol scavenging, impulse spray, electrostatic capture, indoor air purification, Greenfield gap, TiO_2_ sorbent, mathematical modeling, emergency response

## Abstract

The rapid removal of fine contaminant particles (0.1–5 µm), including bioaerosols, from indoor air remains a critical challenge, particularly in emergency scenarios such as industrial accidents or biological incidents. This study develops a novel mathematical model that integrates the kinetic evolution of an impulse-sprayed powder aerosol (as an example, TiO_2_, particle size 5–10 µm) with contaminant-capture efficiency, accounting for Brownian diffusion, inertial impaction, interception, and electrostatic attraction. The model incorporates the triboelectric charge acquired by sorbent particles during pneumatic spraying. Parametric calculations demonstrate that for uncharged particles, capture efficiency in the Greenfield gap is low (η = 0.1–0.3). However, when sorbent particles carry an opposite charge to the contaminants, the efficiency increases to over 0.7, and the characteristic capture time becomes substantially shorter than the gravitational settling time. A model-based criterion for sorption completeness is derived, indicating that, under the specified assumptions, sorbent particles with diameters of 5–7 µm satisfy the criterion at concentrations of approximately 1–2 g/m^3^. The model provides a theoretical and parametric framework for estimating the required sorbent concentration, capture efficiency, and characteristic purification time under specified room and particle conditions. The results can support the preliminary design of rapidly deployable emergency air-cleaning systems.

## 1. Introduction

The problem of indoor air pollution with harmful aerosol particles poses a serious threat to human health [1]. Modern research shows that small particles (i.e., PM_2.5_ and PM_10_) can penetrate the lower respiratory tract. Such particles are dangerous in themselves, but they can also carry viruses and bacteria, facilitating their delivery to the body [2]. The COVID-19 pandemic has demonstrated the critical importance of effective methods for quickly cleaning the air of viral aerosols. Bioaerosols—airborne particles of biological origin—are especially dangerous [3]. They include high-molecular allergens, pollen, pathogenic, and non-pathogenic organisms (e.g., viruses, bacteria, fungi, etc.). Such particles can remain in the air in closed spaces if active measures are not taken to remove them [4]. In addition to biological contaminants, aerosols and toxic industrial dust pose a significant hazard, especially in emergency situations [5,6].

Submicron and micron particles (0.1–5 µm) represent a special risk category [7]. This size range includes most viruses (20–300 nm), bacteria (0.5–5 µm), and fine dust. These particles are the most difficult to remove using traditional methods due to the balance between inertial and diffusion capture mechanisms [8]. The “Greenfield gap” is a well-known range of difficult-to-remove particle sizes—from 0.1 to 2 µm.

In addition to the tasks of continuous air purification in ventilation and air conditioning systems, there are a number of situations requiring rapid intervention. These include industrial accidents with the release of toxic gases and aerosols [9]; terrorist attacks using biological or chemical agents [10]; emergency decontamination of premises upon detection of particularly dangerous infections (including viral and bacterial); and accidents at nuclear power facilities, with the release of radioactive particles [5]. The motivation for the proposed approach is associated with a different operational scenario: rapid, room-scale deployment of a collector aerosol without requiring prior installation of a fixed air-cleaning device.

Modern methods for removing small aerosol particles from the air utilize several classes of technologies: filtration (including HEPA filters), electrostatic precipitators, ultraviolet disinfection, plasma technologies, and wet scrubbing [11,12,13]. However, most of these methods have limitations for emergency use: they require time to set up, are energy-intensive, are not always portable, or have low effectiveness against submicron particles, especially viruses. Traditional spraying of liquids or powders produces particles tens and hundreds of micrometers in size, which quickly settle under gravity and do not have time to effectively interact with small contaminants.

The difficulty of removing submicron particles should, however, be considered in the context of the specific operating principles of each purification technology. In particular, the filtration efficiency of fibrous filters is not monotonically dependent on particle size. Mechanical filtration mechanisms include inertial impaction, interception, and Brownian diffusion, and their relative contributions vary with particle diameter. The particle size corresponding to the minimum filtration efficiency is commonly referred to as the most penetrating particle size (MPPS), which is typically around 0.1–0.3 µm depending on the filter structure and operating conditions. Thus, submicron particles, including particles smaller than the MPPS, are not intrinsically beyond the capability of high-efficiency filtration systems. Rather, the challenge is associated with the minimum of the combined collection mechanisms and with the pressure drop, airflow rate, residence time, and deployment requirements of a particular filtration system [14,15].

Electrostatic precipitation is also an established technology for the removal of fine and submicron particles. Its performance depends on particle charging, electric-field configuration, residence time, and the efficiency of charge transfer to the particles. Recent reviews have demonstrated that electrostatic precipitators can provide high collection efficiencies for nanoparticles and submicron particles, while also identifying particle charging and short residence times as important limitations for compact or rapidly deployable systems [15,16]. Consequently, the present study does not assume that conventional filtration or electrostatic precipitation is intrinsically incapable of removing submicron particles. 

A related class of problems has been studied in aerosol scavenging and electrostatic scrubbing. Classical scavenging models describe particle collection by larger collector particles or droplets through combinations of Brownian diffusion, interception, and inertial impaction. More recent modeling approaches additionally consider electrostatic interactions and other particle-transport mechanisms, particularly in the submicron range [17]. Charged sprays have also been investigated for the removal of submicron particles, and experimental and theoretical studies have demonstrated that electrostatic interactions can substantially enhance collection when collector and contaminant particles have appropriate charge conditions [18]. Similarly, electrostatic attraction and the electrical agglomeration of submicron particles with larger particles have been investigated as a means of increasing subsequent collection efficiency [19].

These studies establish the physical basis for several elements incorporated in the present model, but they generally address different collector configurations and operating regimes. In particular, previous scavenging models have predominantly considered falling hydrometeors or liquid droplets, while electrostatic precipitation models usually consider externally imposed electric fields and stationary collection electrodes. Such formulations do not directly describe the transient evolution of a dispersed solid sorbent cloud in a closed room, in which the collector particles themselves have a finite airborne lifetime determined by gravitational and wall deposition and simultaneously act as mobile collection surfaces for contaminant particles.

The research gap addressed in this work is therefore not the absence of individual models for aerosol sedimentation, particle collision, or electrostatic collection. Rather, it concerns the coupling of these processes for a pulsed solid-phase sorbent aerosol intended for rapid indoor decontamination. To the best of our knowledge, we did not identify a model that simultaneously describes (i) the temporal evolution and sedimentation of a pulsed solid sorbent aerosol in a closed room, (ii) contaminant-particle capture by Brownian diffusion, inertial impaction, interception, and electrostatic interaction, and (iii) the influence of charge acquired by sorbent particles during pneumatic dispersion on the resulting capture kinetics and sorption completeness. The present study therefore combines these previously developed physical descriptions into a unified first-order mathematical framework for emergency indoor aerosol purification.

The proposed framework is intended primarily as a theoretical and parametric model rather than as a universal predictive model for all types of biological, chemical, industrial, or radioactive aerosols. The quantitative calculations presented here use specified representative particle pairs and idealized charge conditions; they should therefore be interpreted as sensitivity analyses of the governing mechanisms rather than as direct predictions for every contaminant class. In particular, transfer of the calculated capture efficiencies between viruses, bacteria, mineral or carbonaceous dust, and radioactive particles requires consideration of their density, morphology, size distribution, hygroscopicity, dielectric properties, surface adhesion, and charge state. Experimental validation for specific sorbent–contaminant pairs remains necessary before the model can be used for engineering design under operational conditions.

An alternative approach is the use of pulsed fine aerosol generators, which create a cloud of submicron- and micron-sized particles that forms in a room within seconds of spraying. This method has previously been shown to be effective for neutralizing harmful gas vapors using metal oxide powders, including titanium dioxide [20]. However, the removal of contaminant particles (as opposed to gases) using pulsed spraying of solid particles has been significantly less studied. The mechanisms of interaction between sorbent particles and harmful aerosols include Brownian diffusion, inertial uptake, interception, and electrostatic attraction, which occur during triboelectrification of particles during spraying [21].

Existing mathematical models in this area can be divided into three areas: (1) models of the evolution of a pulsed aerosol, describing the change in the mass concentration and specific surface area of particles over time [22,23]; (2) models of the capture of contaminant particles by liquid droplets, taking into account various collision mechanisms [21]; and (3) models of the electrostatic charging of particles during pneumatic atomization [24]. The research gap addressed in this work is therefore not the absence of individual models for aerosol sedimentation, particle collision, or electrostatic collection. Rather, it concerns the coupling of these processes for a pulsed solid-phase sorbent aerosol intended for rapid indoor decontamination. The main contribution of the present work is the coupling of these previously established physical descriptions into a single first-order mathematical framework for a pulsed solid-phase sorbent aerosol in a closed indoor space.

The aim of this study is to develop a mathematical model describing the capture of fine contaminant particles (0.1 to 5 µm in size, including viruses, bacteria, and fine dust) by solid-phase sorbent particles instantaneously dispersed in a pulsed manner in a closed space. The model integrates the kinetics of aerosol cloud evolution with the calculation of capture efficiency based on Brownian diffusion, inertial mechanisms, and electrostatic attraction. The study also analyzes the effect of the electric charge of the sorbent particles, generated during dispersal, on the overall air purification efficiency. The developed model is intended as a theoretical framework for evaluating rapid-response aerosol purification scenarios in industrial, public, laboratory, and medical facilities. Its practical implementation requires additional assessment of aerosol exposure, sorbent safety, ventilation, post-treatment removal, and conditions for safe re-occupation of the treated space.

The model is generic at the level of particle-transport and capture mechanisms, whereas the numerical results are system-specific. To obtain numerical results in a particular system, the particle density, particle morphology/sphericity, particle-size distribution, dielectric permittivity, surface charge, hygroscopicity, adhesion characteristics, and sorbent–contaminant material pair must be known.

## 2. Method, Problem Statement and Basic Equations

The method used in this study is mathematical modeling. The assumptions adopted in the model are formulated below.

Assume that an aerosol consisting of harmful particles (contaminant particles) with a characteristic size of *D_pc_* ~ 0.1–5 μm is uniformly distributed in the air of a room. We will use the Sauter diameter as the characteristic size. Another aerosol consisting of sorbent particles with a characteristic size of *D_sp_* ~ 5–10 μm is instantly sprayed into the air and uniformly distributed in space. The mass concentrations of the first and second aerosols are known: *C_pc_* and *C_sp_*, respectively. The sorbent particles have an electrostatic charge acquired during spraying, while the contaminant particles have no charge or the opposite charge. The aerosol particles will slowly settle under the force of gravity, interacting with each other. The sorbent particles will capture and retain the contaminant particles on their surfaces due to electrostatic and van der Waals interactions. The modeling task is to answer the question of how effectively contaminant particles are removed by sorbent particles during the time the sorbent particles are present in the air (before they settle).

As noted in the Introduction, we will break this problem down into three subproblems and combine them.

### 2.1. Change in the Mass of Aerosol Particles in the Air

A method of pulsed aerosol spraying using HEM energy is known. With this method, a uniformly distributed aerosol cloud is formed in the room volume within a few seconds due to turbulent/convective diffusion [25]. Thus, we will assume that at the initial moment of time, two types of aerosols are present in the room. Particles of these aerosols will slowly settle under the influence of gravity, and also settle on the walls of the room. Analytical expressions for estimating the dynamics of change in the mass of aerosols due to sedimentation on the bottom and walls of the room with a characteristic size *H* are obtained in [22,23]. In the general case, for the mass of settled particles *m_p_* at time *t*, we have:

(1) $$\frac{m_{p}(t)}{m_{p}(0)}=1-\exp\left(-\gamma t\right)$$

where γ is the settling velocity coefficient, which depends nonlinearly on the particle diameter:

(2) $$\gamma (D_{p})=\frac{u_{s}}{H}+\beta =\frac{\rho_{p}D_{p}^{2}}{18\mu H}g+\frac{\beta_{0}k_{D0}}{H}\frac{T}{\mu D_{p}}$$

Here, *u_s_* is the Stokes sedimentation velocity,

(3) $$u_{s}=\rho_{p}D_{p}^{2}g/18\mu$$

where ρ*_p_* is the particle density and *g* is the acceleration of free fall. The deposition coefficient on the walls β is proportional to the diffusion coefficient and inversely proportional to the distance to the walls:

(4) $$\beta =\frac{\beta_{0}k_{D}}{H}$$

where β_0_ is a free parameter of the model [1/m]. The diffusion coefficient *k_D_* [N m/K] is proportional to the absolute temperature *T*, and inversely proportional to the viscosity of air μ and the characteristic particle diameter *D_p_*:

(5) $$k_{D}=\frac{k_{D0}T}{D_{p}\mu }$$

where the coefficient *k_D_*_0_ is a free parameter of the model. In the ideal case of Brownian diffusion in the absence of convective/turbulent flows, the coefficient *k_D_*_0_ is approximately equal to the Boltzmann constant divided by 3π, but in real conditions it is greater. In [23], the coefficients for pulsed sputtering of titanium oxide were determined experimentally: β_0_ = 0.025 1/m, *k_D_*_0_ = 3 × 10^−15^ N m/K.

There is a diameter corresponding to the minimum of the function γ(*D_p_*):

(6) $$D_{\min}=\sqrt[{3}]{\frac{9\mu \beta_{0}k_{D0}}{\left(\rho_{p}-\rho \right)g}}$$

This diameter corresponds to the case where the aerosol remains in the air for the maximum possible time, without settling on the walls or bottom of the chamber.

Expressions (1–6) apply to both the sorbent aerosol and the contaminant aerosol, with the corresponding diameter and particle density of these aerosols. When describing the contaminant aerosol, we will add the subscript *c* to the variables, and for the sorbent aerosol, the subscript *s*. Also, to avoid confusion, we will refer to contaminant particles as PCs, and sorbent particles as PSs. At each time *t*, the mass of contaminant particles remaining in the air is *m_pc_*(0)—*m_pc_*, the mass of sorbent particles is *m_ps_*(0)—*m_ps_*, and the mass concentration of contaminant and sorbent particles is, respectively:

(7) $$\begin{array}{l} C_{pc}=C_{pc}(0)\exp\left(-\gamma (D_{pc})t\right)\\ C_{ps}=C_{ps}(0)\exp\left(-\gamma (D_{ps})t\right) \end{array}$$

These particles will interact with each other. We are interested in the capture of contaminant particles by sorbent particles.

### 2.2. Capture of Contaminant Particles by Sorbent Particles

We define the capture efficiency η as the ratio of the number of PCs actually captured by a PS to the number of PCs that could have collided with the PS if they had moved in a straight line within the cross-sectional projection of the PS. Next, we present formulas for the components of the PC capture efficiency by PSs, corresponding to different capture mechanisms.

#### 2.2.1. Inertial Capture (Particles Deflect from the Streamlines and Collide with the Droplet)

In our problem, the particles of the sprayed sorbent aerosol are somewhat larger than the contaminant particles. Therefore, the gravitational settling velocity for them is different. The relative gravitational settling velocity *U* between PSs and PCs is determined by the expression:

(8) $$U=\frac{g}{18\mu }\left(D_{ps}^{2}\rho_{ps}-D_{pc}^{2}\rho_{pc}\right)$$

The efficiency is determined by the Stokes number Stk, written taking into account expression (4) for the relative velocity of particles:

(9) $$\mathrm{Stk}=\frac{\rho_{pc}D_{pc}^{2}U}{18\mu D_{ds}}$$

When writing the Stokes number, we consider a PC colliding with an obstacle in the form of a PS in a flow of velocity *U*. The dependence of the inertial capture component for three ranges of the Stk number is known [26]:

(10) $$\eta_{iner}=\left\{\begin{array}{l} 0,\;\mathrm{Stk}\leq 0.083\\ 8.57{\left(\frac{\mathrm{Stk}}{\mathrm{Stk}\;+\;0.5}\right)}^{2}(\mathrm{Stk}-0.083),\;0.083<\mathrm{Stk}<0.2\\ {\left(\frac{\mathrm{Stk}}{\mathrm{Stk}\;+\;0.5}\right)}^{2},\;\mathrm{Stk}\geq 0.2 \end{array}\right.$$

#### 2.2.2. Diffusion Capture (Particles Move Randomly Due to Brownian Diffusion)

The chaotic thermal motions of particles of both types increase the probability of their collisions. The capture efficiency depends on the Peclet number [27]:

(11) $$\eta_{dif}=4{\mathrm{Pe}}^{-2/3},\;\mathrm{Pe}=\frac{D_{ps}U}{k_{D}}$$

#### 2.2.3. Interception (PCs Follow the Current Lines and “Grab” the PS if They Are Close Enough)

In this case, the particle can be captured due to adhesion on the sorbent surface if the current line which passes at a distance from the PS surface is less than or equal to the particle radius of the PC. The interception efficiency depends on the ratio of the particle sizes [28,29]:

(12) $$\eta_{inter}\approx 3/2{\left(\frac{D_{pc}}{D_{ps}}\right)}^{2}$$

#### 2.2.4. Electrostatic Capture

Virus envelopes and bacterial cell walls often have a negative charge due to ionized groups on the surface. Under typical air conditions, the charge remains negative. For example, for SARS-CoV-2 it ranges from −10 to −50 mV. If the sorbent aerosol particles are electrostatically neutral, then they can be polarized in the presence of a charged bioaerosol PC. The negative charge of the particle repels electrons in the sorbent particle, creating a local positive charge on its surface, which leads to attraction. Below, we will consider the case of when the sorbent particles have an electrostatic charge obtained when passing through the spray nozzle. In this instance, they will be able to similarly attract electrically neutral (or oppositely charged) particles of contaminants. In any case, for aerosols of high dispersion and a sufficiently high volume concentration of particles, it is necessary to take into account the electrostatic component of capture.

For a charged particle with charge *q* and a neutral particle, the force of attraction in the approximation of a point charge and a polarizable sphere is given as:

(13) $$F_{electr}=\frac{q^{2}D_{ps}^{3}}{32\pi \varepsilon_{0}r^{4}}\frac{\varepsilon -1}{\varepsilon +2}$$

where *r* is the distance between the center of the PCs and PSs, ε_0_ is the electric constant, ε is the permittivity of the PS, and (ε − 1)/(ε + 2) is the polarization factor. This force decays as 1/*r*^4^, making it significant only at short distances. The particle’s charge depends on its surface area and is therefore proportional to the square of its diameter (*q*_0_ is the proportionality constant). The electrostatic contribution to the capture efficiency depends on the dimensionless parameter *N_E_*, which characterizes the ratio of the electrostatic force to the resistance of the medium:

(14) $$\eta_{elec}\sim N_{E}=q_{0}D_{pc}^{2},\;\;N_{E}=\frac{q^{2}}{3\pi^{2}\varepsilon_{0}\mu UD_{ps}^{2}}$$

Viruses and bacteria can carry an electric charge due to the ionization of surface groups or the adsorption of ions. For viruses with a diameter of 20–200 nm, the charge is 10–100 e, while for bacteria with a diameter of 0.5–10 μm, it is 10^4^–10^6^ e [30,31]. This allows us to estimate the coefficient *q*_0_.

The adopted values of the surface charge density coefficient *q*_0_ ≈ 2 µC m^−2^ for contaminant particles and the scaling *q_ps_* =10^−12^*D_ps_*^2^ C for sorbent particles are order-of-magnitude estimates based on reported charges of bioaerosols [30,31] and triboelectric charging during pneumatic dispersion of metal oxide powders [24,32,33]. Actual charges can vary substantially depending on material pair, nozzle geometry and material, relative humidity, aerosol aging, and the presence of free ions. High relative humidity generally accelerates charge dissipation, while forced charging of the nozzle (when feasible) can increase the specific charge by one to two orders of magnitude. In the present parametric study, these values are treated as constant; the influence of their uncertainty is examined in Section 3.1 by varying *q*_0_ and *q_ps_* over physically plausible ranges. Charge relaxation and environmental effects are therefore acknowledged as model limitations and are left for future experimental refinement.

### 2.3. Charge of Sorbent Particles and Electrical Capture Component with Opposite Particle Charges

Experiments involving the atomization of various materials through a nozzle indicate the charge acquired by particles after passing through the nozzle as a result of friction [24]. Electrons are transferred when particles rub against the nozzle walls, the atomizer channels, or each other. The sign and magnitude of this charge depend on the difference in the triboelectric series of the materials. For example, titanium dioxide particles often acquire a positive charge upon exiting a metal nozzle (while silicon oxide particles, conversely, acquire a negative charge). Typical charges are *q* ≈ 10^−15^ − 10^−12^ C per 1–50 μm particle.

This corresponds to a specific charge of 10^−6^ to 10^−3^ C/kg. Smaller particles acquire a higher specific charge due to their large specific friction surface area. Clearly, forced charging (when the nozzle is energized) allows for a charge level several orders of magnitude higher than that achieved by friction alone—up to 0.1–2.3 μC/kg [32,33]. It is also possible to control the particle charge sign by varying the voltage on the electrode (the particles will acquire the opposite charge sign). This is especially valuable if the goal is to collect contaminant particles with a predetermined charge sign (e.g., viruses and bacteria with a negative charge sign). The dielectric properties of the particles determine the ability to retain the acquired charge [34].

If the contaminant and sorbent particles are oppositely charged, the electrical capture mechanism becomes significantly stronger than in the case of a neutral particle of one type and a charged particle of another type (induced attraction). In this case, a direct Coulomb force of attraction (not just polarization) acts between the particles. This force decays as 1/*r*^2^, but at relatively large distances (several contaminant particle diameters), it can significantly deflect the particle’s trajectory toward the sorbent particle. As a result, the sorbent is capable of capturing particles even from trajectories that would otherwise pass by without an electric field (the effective capture area can be significantly larger than the geometric cross-section of the sorbent particle). The interaction force is determined as follows:

(15) $$F_{electr}=\frac{\left|q_{pc}q_{ps}\right|}{4\pi \varepsilon_{0}r^{2}}$$

where *q_pc_* and *q_ps_* are the charges of the contaminant and sorbent particles, respectively. Then, the electrical capture component and the dimensionless parameter *N_E_* will take the form [26,35]:

(16) $$\eta_{elec}\approx \frac{N_{E}}{N_{E}+0.5},\;N_{E}=\frac{\left|q_{pc}q_{ps}\right|}{3\pi^{2}\varepsilon_{0}\mu UD_{ps}^{2}}$$

It can be expected that the electrical component of the sorbent’s capture of contaminant particles will be quite significant. Furthermore, the opposite charge of the particles will help not only to capture but also to retain harmful particles on the sorbent’s surface.

### 2.4. Correction for Small Particles

For relatively large particles (compared to the mean free path of molecules), the continuous medium approximation and the Stokes equation are valid. The applicability of this approximation is determined by the Knudsen number:

(17) $$\mathrm{Kn}\;=\;\frac{2\lambda }{D_{pc}}$$

where λ is the mean free path of air molecules (~68 nm under normal conditions). For particles with a diameter much larger than λ, the condition Kn << 1 is satisfied. However, for particles of 10–100 nm (e.g., viruses), Kn ~ 1, and the continuous medium approximation begins to break down. Then, the diffusion coefficient for such small particles is determined with the Cunningham correction [36]:

(18) $$k_{D1}\;=\;k_{D}C_{K},\mathrm{where}\;C_{K}=\left(1+1.257\mathrm{Kn}+0.4\exp(-1.1/\mathrm{Kn})\right)$$

The particle settling velocity can also be determined with this correction:

(19) $$U=\frac{g}{18\mu }\left(D_{ps}^{2}\rho_{ps}-D_{pc}^{2}\rho_{pc}C_{K}\right)$$

### 2.5. Relative Mass of Captured Contaminant Particles and Characteristic Capture Time

The overall efficiency of particle capture by a sorbent is described by the capture coefficient η, which depends on the interaction mechanism. The overall capture coefficient can be written as a combination of different mechanisms, taking into account their independence:

η = 1 − (1 − η_iner_)(1 − η_dif_)(1 − η_inter_)(1 − η_elec_) (20)

The capture efficiency η determines the fraction of contaminant particles that collide with a sorbent particle from the flow passing through the cross-section of the PS. The flux of particles captured by the sorbent is proportional to the cross-sectional area of the PS, the relative flow velocity, and the particle concentration. The rate of change in the mass concentration of PCs *m_pc_* is determined by their capture by PSs; it is proportional to the concentration of both particles, as well as the flux of PCs captured by the sorbent.

The change in the concentration of captured contaminant particles over time is determined by a system of equations:

(21) $$\left\{\begin{array}{l} \frac{dC_{pc}}{dt}=-\left(k_{capt}+\gamma_{pc}\right)C_{pc}\\ \frac{dC_{ps}}{dt}=-\gamma_{ps}C_{ps} \end{array}\right.$$

where the capture constant is $k_{capt}=\frac{3}{2}\frac{U\eta }{\rho_{ps}D_{ps}}C_{ps}$ The first equation is written for contaminant particles and takes into account both their capture by the sorbent and sedimentation. The second equation is written for sorbent particles and takes into account sedimentation only. If the change in concentration due to sedimentation during the capture time can be neglected, we obtain an analytical solution for the contaminant particles remaining in the air, as follows:

(22) $$C_{pc}=C_{pc}(0)\left(1-\exp\left(-k_{capt}t\right)\right)$$

The characteristic time of particle capture is then as follows:

(23) $$t_{capt}=\frac{1}{k_{capt}}=\frac{2}{3}\frac{D_{pc}\rho_{pc}}{U\eta C_{ps}(0)}$$

The characteristic capture time *t_capt_* is defined as the time at which the contaminant concentration would decrease by a factor of e if the sorbent concentration remained constant and sedimentation were neglected. It therefore provides a convenient order-of-magnitude measure of the intrinsic speed of the capture process under given particle and concentration conditions. It is also useful to compare the characteristic capture time with the characteristic settling time of particles of both types:

(24) $$\begin{array}{l} t_{depc}=\frac{1}{\gamma (D_{pc})}=\frac{18\mu HD_{pc}}{\rho_{pc}D_{pc}^{3}g+18\beta_{0}k_{D0}T}\\ t_{deps}=\frac{1}{\gamma (D_{ps})}=\frac{18\mu HD_{ps}}{\rho_{ps}D_{ps}^{3}g+18\beta_{0}k_{D0}T} \end{array}$$

A comparison of *t_capt_* with the characteristic sedimentation times *t_deptc_* of the contaminant particles is used in this work as a theoretical indicator. When *t_capt_* < *t_deptc_*, capture is expected to dominate over gravitational settling within the idealized framework of the model. Conversely, when the two times are comparable or *t_capt_* > *t_deptc_*, a substantial fraction of particles may deposit on surfaces before being captured. It should be emphasized that these characteristic times are not deterministic removal times. In a real aerosol population, the processes of capture, sedimentation, turbulent mixing, wall deposition, and (possibly) aggregation occur simultaneously and are inherently probabilistic. The comparison of characteristic times therefore serves only as a first-order diagnostic of the relative importance of the competing mechanisms under the model assumptions; quantitative predictions of residual airborne concentrations require solution of the full system of Equation (21) (or more detailed numerical models).

### 2.6. Model Assumptions and Limitations

The mathematical formulation relies on a number of simplifying assumptions that enable analytical and semi-analytical treatment while limiting the direct quantitative applicability of the results to real indoor environments.

First, both the contaminant aerosol and the pulsed sorbent aerosol are assumed to be spatially uniform at the initial instant and to remain well mixed thereafter. In practice, emergency scenarios may involve localized sources, incomplete turbulent mixing, ventilation-driven advection, thermal stratification, and deposition on complex room surfaces. These effects can produce spatial heterogeneity in particle concentrations and reduce the effective contact time between sorbent and contaminant particles relative to the idealized case.

Second, particle populations are represented by characteristic (Sauter-mean) diameters rather than full size distributions. Polydispersity can alter the relative importance of diffusion, interception, and inertial mechanisms and can change the effective capture efficiency and sedimentation rates. The present monodisperse treatment therefore provides first-order estimates that should be refined by moment or sectional methods when detailed size spectra are available.

Third, the model neglects inter-particle coagulation of sorbent particles, charge dissipation (or acquisition) over time, hygroscopic growth, ventilation, and air exchange. Charge magnitudes are prescribed on the basis of literature values and typical triboelectric charging during pneumatic dispersion; they are treated as constant. Relative humidity, surface conductivity, and aerosol aging can substantially modify electrostatic interactions in real rooms.

Fourth, the capture efficiency is constructed by combining independent mechanism efficiencies (Equation (20)). While this is a standard engineering approximation, the simultaneous action of several mechanisms and hydrodynamic interactions at high concentrations may introduce corrections that are not captured here.

Consequently, the calculated capture efficiencies, characteristic times, and minimum sorbent concentrations should be interpreted as theoretical indicators under the stated idealizations rather than as precise predictions for an arbitrary indoor emergency. Experimental validation for specific sorbent–contaminant pairs and more detailed simulations that incorporate spatial heterogeneity remain necessary before the model can be used for quantitative engineering design.

Table 1 shows the main parameters of the model and their values.

The mathematical structure of the model is generic with respect to the underlying transport and capture mechanisms. Quantitative application to a different sorbent–contaminant system requires only the substitution of the corresponding particle properties (density, characteristic size or size distribution, dielectric permittivity, surface charge, etc.). The numerical values reported in Section 3 are therefore illustrative for the chosen parameter sets rather than universal constants.

## 3. Calculation Results and Discussion

The mathematical model developed above enables the evaluation of contaminant-particle capture by a pulsed sorbent aerosol. A parametric study is therefore performed to identify favorable conditions for capture.

### 3.1. Components of Capture Efficiency Depending on Particle Size

**Figure 1:**
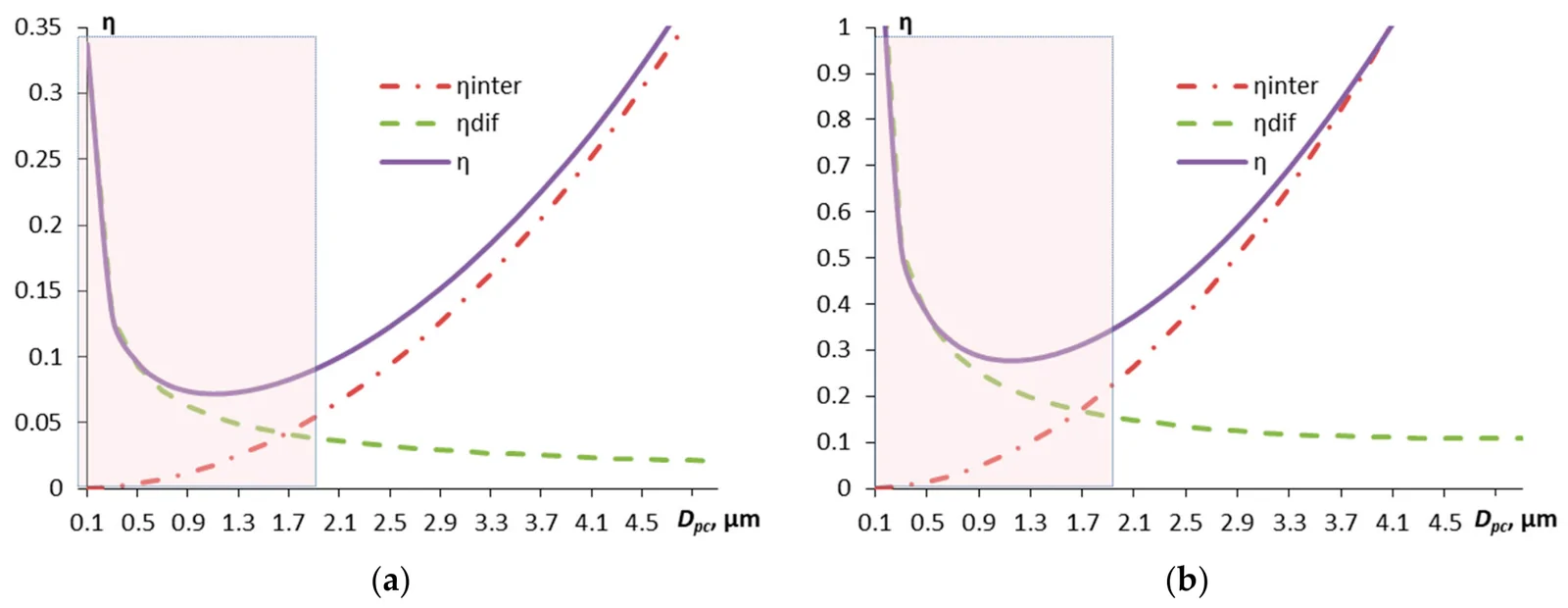
Components of the capture efficiency η of contaminant particles by sorbent particles as a function of contaminant diameter *D_pc_*. Sorbent diameter *D_ps_* = 10 μm (**a**); *D_ps_* = 5 μm (**b**). Calculations were performed for uncharged particles (carbon contaminant, TiO_2_ sorbent). Solid, dashed, and dash-dotted lines show the contributions of interception, Brownian diffusion, and inertial impaction, respectively; the thick solid line is the overall efficiency (Equation (20)). The shaded region indicates the Greenfield gap (approximately 0.1–2 µm).

Figure 1 shows the efficiency of contaminant particle-capture by sorbent particles as a function of particle size. The calculations assume that the contaminants are carbon particles, and the sorbent is titanium oxide. Particle charge is neglected. In this case, contaminant particle-capture by the sorbent occurs almost exclusively through the interception mechanism. Diffusion interaction is effective only for particles smaller than 0.5 µm, and the inertial component is small due to the small difference in particle size between the PSs and PCs. Smaller sorbent particles capture contaminants with a diameter of 3.7 µm or more with an efficiency of η = 1. However, in the Greenfield gap, capture efficiency is low: η ~ 0.1–0.3.

**Figure 2:**
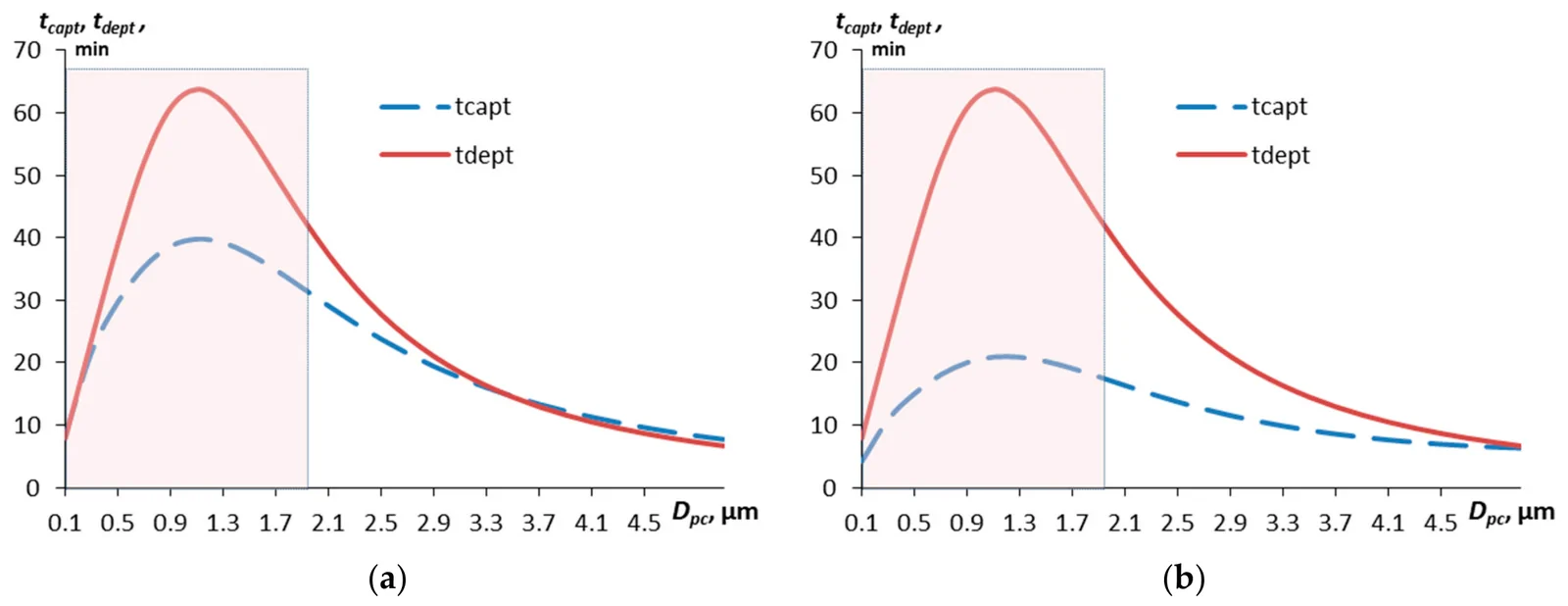
Characteristic capture time *t_capt_* and sedimentation time *t_dept_* of contaminant particles versus contaminant diameter: *D_pc_*:*D_ps_* = 10 μm (**a**); *D_ps_* = 5 μm (**b**). Sorbent concentration *C_ps_* = 1 g m^−3^, room height *H* = 5 m; particles are uncharged. The shaded region indicates the Greenfield gap.

We will estimate the lifetime of contaminant and sorbent aerosols before precipitation and the characteristic capture time, according to expressions (23) and (24) (Figure 2). The calculation assumes a constant sorbent particle concentration of 1 g/m^3^ and a typical room size of *H* = 5 m.

Particle capture time is shorter than settling time across virtually the entire particle size range, indicating that, within the model framework, capture is expected to proceed faster than gravitational settling. The Greenfield gap coincides with the maximum of both the sedimentation and capture times: particles in this size range remain airborne longest and interact less effectively with sorbent particles. Furthermore, the lifetime of heavier sorbent particles is shorter than that of contaminant particles (approximately 29 min for 5 µm versus 8 min for 10 µm sorbent particles). This suggests that larger sorbent particles may not remain airborne long enough to capture the entire contaminant load under the assumed conditions.

**Figure 3:**
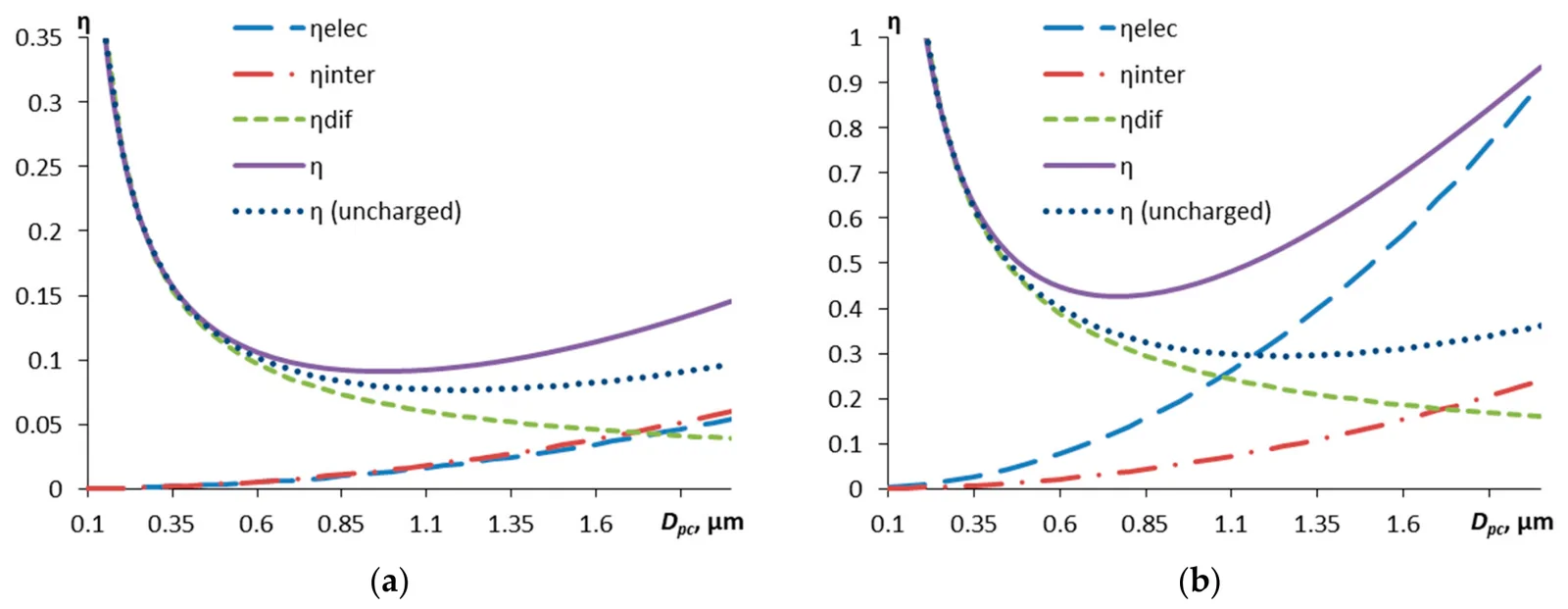
Overall capture efficiency η versus contaminant diameter *D_pc_* when only the contaminant particles are charged. *D_ps_* = 10 μm (**a**); *D_ps_* = 5 μm (**b**). Solid lines—calculation with charge (*q*_0_ = 2 µC m^−2^); dotted lines—uncharged case.

The preceding calculations neglected the electrical charge of the particles. To assess the influence of charge, the calculations were repeated for electrically neutral sorbent particles and negatively charged contaminant particles (bacteria or viruses, 0.1–2 µm) with a surface charge density coefficient of *q*_0_ = 2 µC m^−2^ at a sorbent concentration of 1 g m^−3^. A correction for small particles (Equations (16)–(19)) was included. The results are shown in Figure 3.

As the calculations show, charged particles are captured much more efficiently by sorbent particles, especially in the contaminant particle size range of ~0.5 µm. As in the previous calculation, smaller sorbent particles capture contaminant particles better. Sorbent particles of characteristic size 5 µm in this case partially mitigate the low capture efficiency associated with the Greenfield gap.

**Figure 4:**
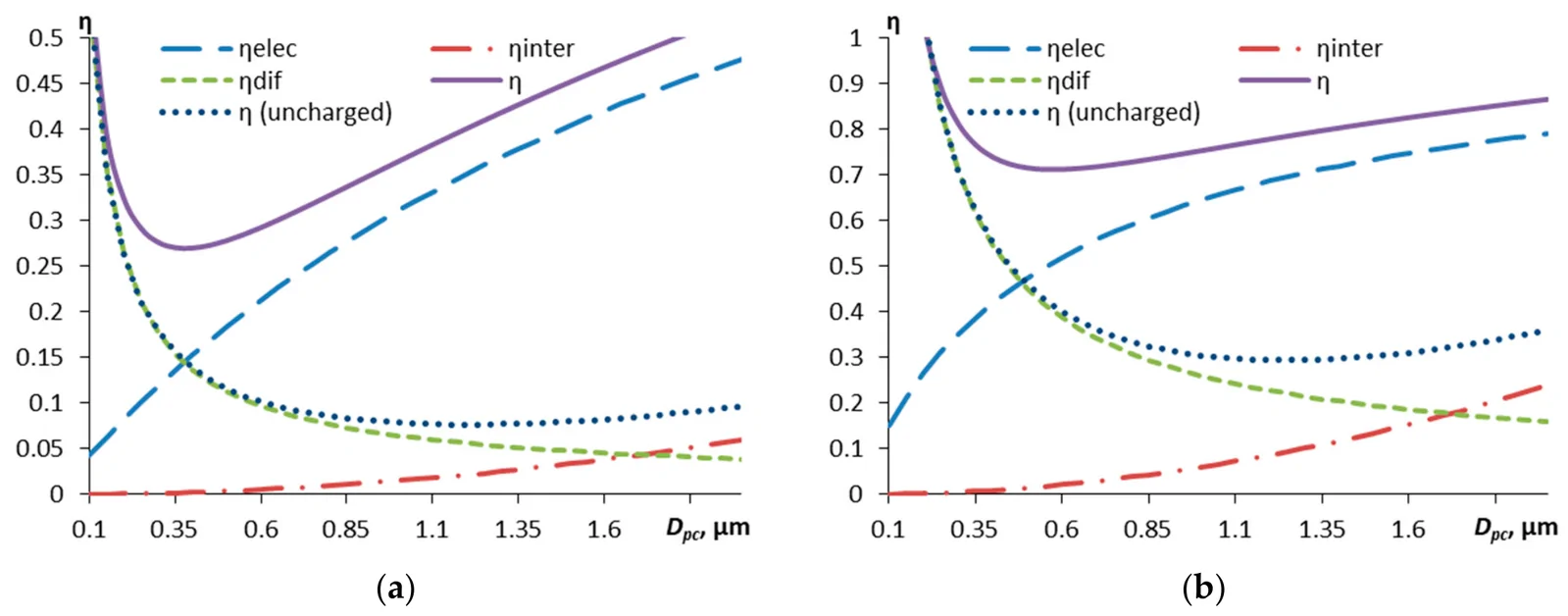
Overall capture efficiency η versus contaminant diameter *D_pc_* when contaminant and sorbent particles carry opposite charges: *D_ps_* = 10 μm (**a**); *D_ps_* = 5 μm (**b**). Solid lines—calculation with opposite charges (*q_ps_* =10^−12^ *D_ps_*^2^ C); dotted lines—uncharged case.

In the following calculations, we assume that the sorbent particles have a charge opposite to that of the contaminant particles. This further increases the capture efficiency (Figure 4). In the calculations, we assume that the *q_ps_* = 10^−12^ *D_ps_*^2^ Cl.

**Figure 5:**
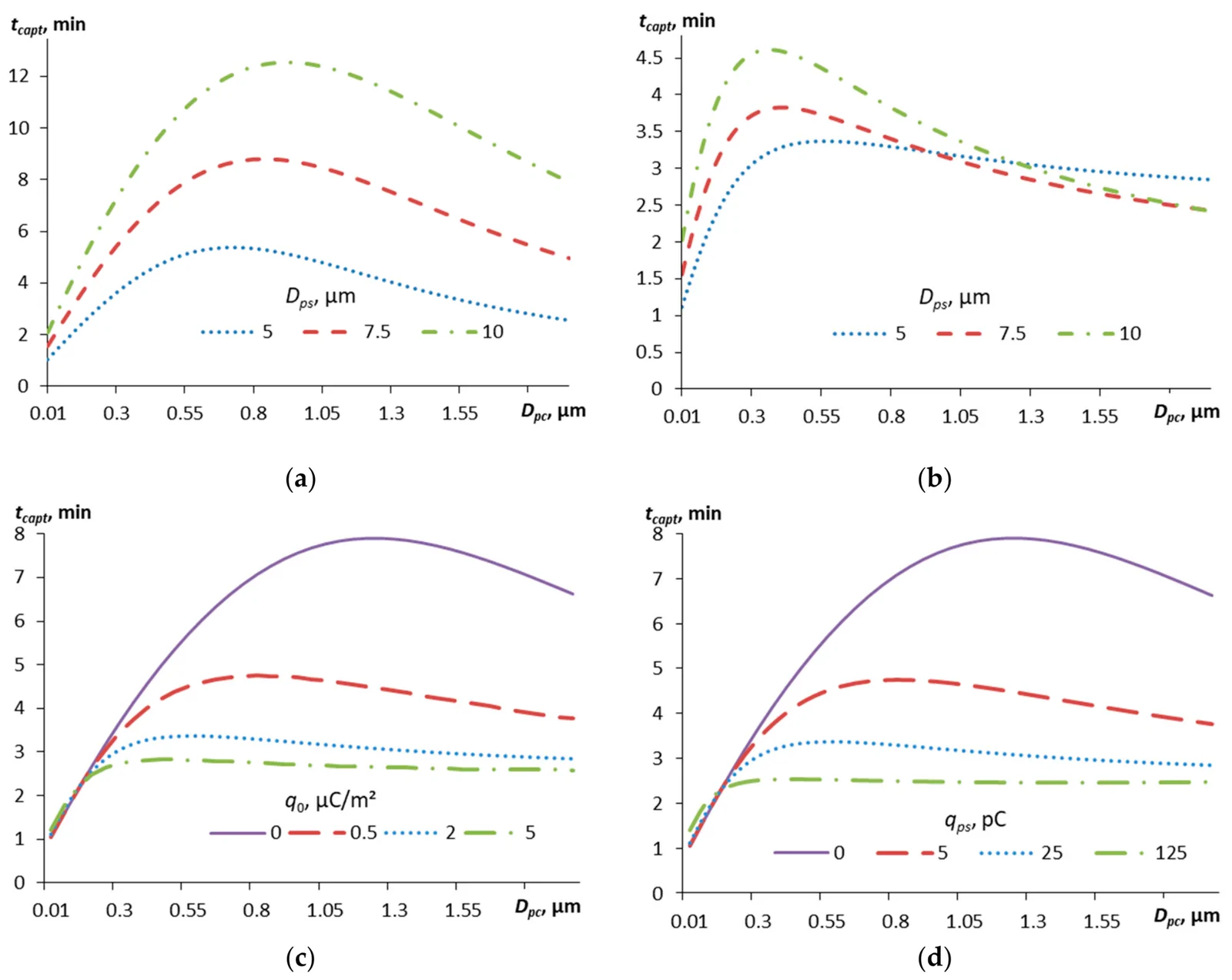
Characteristic capture time *t_capt_* of contaminant particles versus contaminant diameter *D_pc_*. Only contaminant particles are charged (**a**); contaminant and sorbent particles have opposite charges (**b**); sensitivity to the surface charge density coefficient of contaminant particles *q*_0_ (0.5, 2 and 5 µC m^−2^) at fixed sorbent charge (**c**); and sensitivity to the charge of sorbent particles *q_ps_* (5, 25 and 125 pC) at fixed contaminant charge (**d**). Calculations for panels (**c**,**d**) were performed at *D_ps_* = 5 µm and *C_ps_* = 1 g/m^3^.

The capture efficiency component due to the opposite charges of the sorbent and contaminant particles increases in the Greenfield gap, compensating for the decrease in the capture component due to diffusion. For small sorbent particles (5 µm in size), the total capture efficiency does not fall below 0.7 and then increases again toward 1 as the contaminant particle size increases. Figure 5 shows the characteristic capture times for contaminant particles for the case of only PCs being charged, and for the case of oppositely charged particles of both types.

Note that the particle capture time in the Greenfield gap in the presence of a charge (especially if both types of particles are oppositely charged) is much shorter than the particle settling time (Figure 2). Therefore, within the idealized model, capture is expected to dominate over sedimentation for contaminant particles in this size range. Even with a 2–5-fold reduction in charge, the efficiency in the Greenfield gap remains significantly higher than in the absence of charge.

Because the principal improvement of capture efficiency inside the Greenfield gap is attributable to the electrostatic contribution, a local sensitivity analysis with respect to the charge parameters was performed. Figure 5c,d shows the characteristic capture time *t_capt_* as a function of contaminant diameter for several values of the surface charge density coefficient *q*_0_ (while the sorbent charge scaling is held fixed) and, separately, for different *q_ps_*. Even when *q*_0_ is reduced by a factor of four relative to the baseline value, *t_capt_* in the Greenfield gap remains several times shorter than the corresponding sedimentation time, and the overall capture efficiency stays well above the uncharged case. Conversely, higher charges further shorten the capture time. These results indicate that the qualitative conclusion—that opposite charging substantially mitigates the Greenfield-gap limitation—is robust within the examined range of electrostatic parameters. Quantitative predictions for a specific material pair will, of course, require experimentally measured charges under the relevant spraying and environmental conditions.

The magnitude and trends obtained in the present calculations are broadly consistent with previously reported findings on aerosol scavenging and electrostatic collection. Classical and more recent studies of particle collection by larger collectors (droplets or solid particles) show that mechanical capture efficiencies in the Greenfield gap are typically low (often well below 0.5) in the absence of electrostatic forces, while opposite charging or imposed electric fields can raise collection efficiencies substantially, sometimes by factors of several times [14,17,18,19,26,35]. The characteristic residence times of micron-sized particles obtained here (tens of minutes for 5 µm sorbent particles in a room of height ~5 m) are also consistent with order-of-magnitude estimates based on Stokes settling velocities reported in standard aerosol literature [26]. These qualitative agreements provide an external plausibility check for the model behavior, although quantitative differences are expected because previous studies mostly considered continuous sprays, falling hydrometeors, or externally imposed electric fields rather than a pulsed solid-phase sorbent cloud in a closed room.

### 3.2. Particle Concentration and Sorption Completeness Criterion

The effectiveness of sorbent particles to capture contaminant particles depends not only on the capture efficiency parameter but also on the concentration of particles of both types and their ratio. If the characteristic capture time is substantially shorter than the characteristic settling time, this is taken as an indication that capture is likely to dominate over deposition under the model assumptions. However, the sorbent concentration may not be sufficient to capture all contaminant particles.

The effectiveness of sorbent particles in capturing contaminant particles depends not only on the capture efficiency parameter but also on the concentrations of both particle populations and their ratio. If the characteristic capture time of a contaminant particle by the sorbent is shorter than the characteristic settling time, the model predicts that capture by the sorbent is favored over direct deposition. However, this condition alone does not ensure that the entire contaminant population is captured because the available sorbent concentration may be insufficient.

To quantify this effect, we introduce a model-based criterion for sorption completeness. The criterion is derived from the ratio of the characteristic settling time of the sorbent particles to the characteristic time associated with contaminant sedimentation and capture. It therefore represents a theoretical threshold for the specified particle properties, concentrations, and model assumptions, rather than a guarantee of complete purification under real indoor conditions.

Within this framework, the calculated minimum sorbent concentration *C_ps_*_min_ should be interpreted as the minimum theoretical concentration predicted by the model to satisfy the sorption-completeness criterion for the specified conditions. The criterion does not account for spatial concentration gradients, ventilation and air exchange, aerosol polydispersity, particle aggregation, or other processes that may occur in realistic indoor environments.

**Figure 6:**
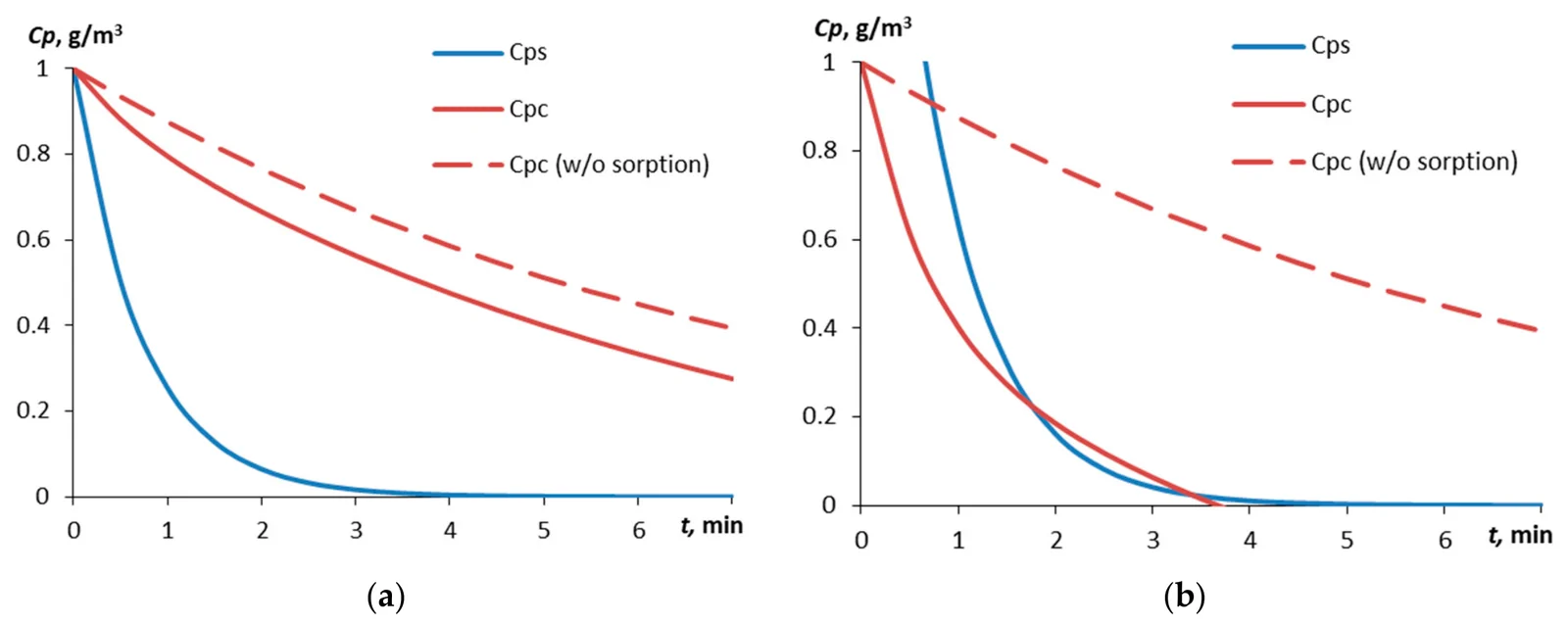
Time evolution of the mass concentrations of sorbent *C_ps_* and contaminant *C_pc_* particles. Initial sorbent concentration *C_ps_*(0) = 1 g/m^3^ (**a**); *C_ps_*(0) = 2 g/m^3^ (**b**). Solid lines—full solution of the kinetic system (Equation (21)) including capture; dashed line—sedimentation of contaminant particles in the absence of sorption. Particle diameters: *D_ps_* = 10 µm, *D_pc_* = 5 µm; initial contaminant concentration *C_pc_*(0) = 1 g m^−3^.

The solution to the system (21) shows the dynamics of the decrease in particle concentrations, taking into account their sorption. Let us consider an example calculation for relatively large particles—*D_ps_* = 10 μm, *D_pc_* = 5 μm. In the calculation, the initial concentration of contaminant particles is 1 g/m^3^. These particles are coal dust, and the sorbent particles are titanium oxide. Figure 6 shows the calculated dynamics of the change in the concentration of particles of both types.

In the first case, when the sorbent particle concentration *C_ps_*(0) = 1 g/m^3^, these particles settle on surfaces faster than they can collect all the contaminant particles. The difference in the contaminant particle concentrations with and without sorption is insignificant (approximately 10%). If the sorbent concentration is twice as high, *C_ps_*(0) = 2 g/m^3^, then the sorbent particles will collect all the contaminant particles before settling.

The criterion for assessing the completeness of particle sorption can be the ratio of the characteristic times of sorbent sedimentation and sedimentation + capture of contaminant particles:

(25) $$\tau_{full}=\frac{\gamma (D_{pc})+k_{capt}(C_{ps}(0))}{\gamma (D_{ps})}\geq 1$$

From this condition, we obtain an expression for the model-based minimum sorbent concentration:

(26) $$C_{ps}(0)\geq C_{ps\min}=\left(\gamma (D_{ps})-\gamma (D_{pc})\right)\frac{2D_{ps}}{3U\eta }$$

The resulting *C_ps_* _min_ should therefore be interpreted as a theoretical threshold corresponding to τ*_full_* ≥ 1 under the specified model assumptions.

**Figure 7:**
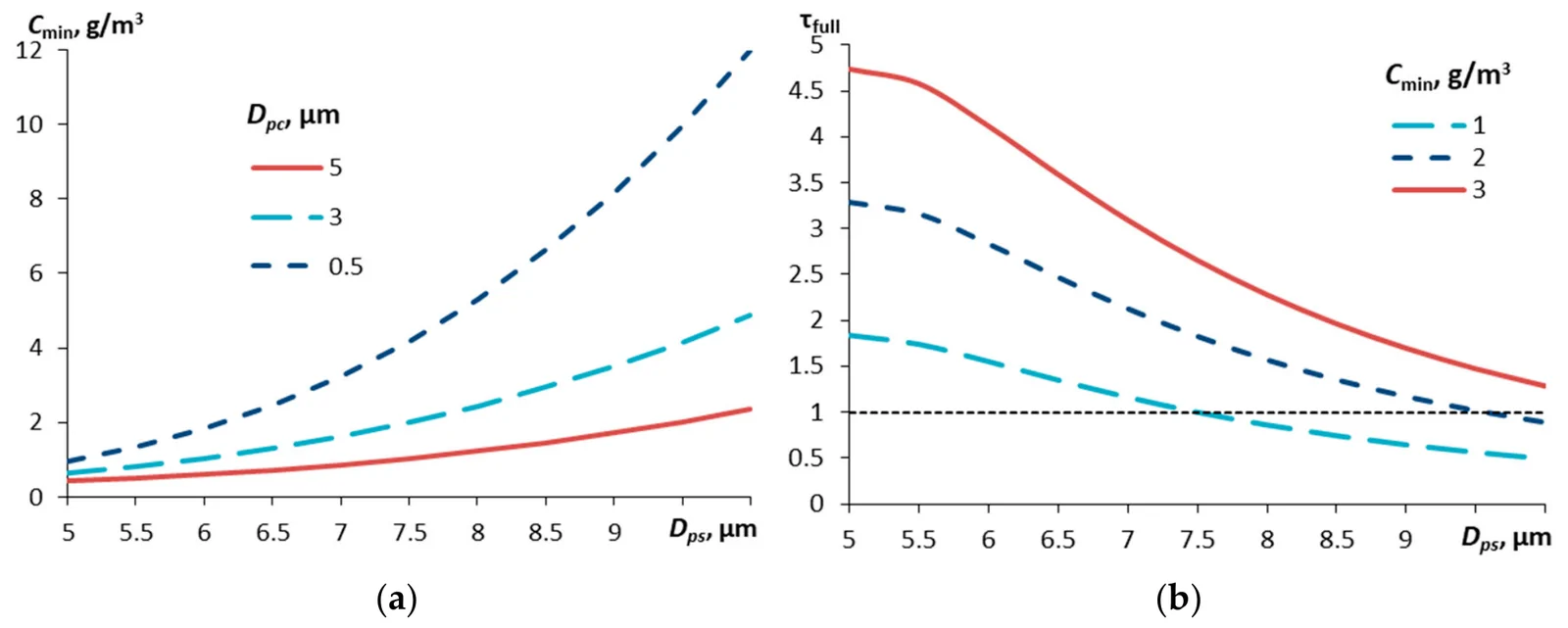
Minimum sorbent mass concentration *C_ps_* _min_ required for essentially complete contaminant collection (under the model assumptions) as a function of sorbent diameter *D_ps_* for selected contaminant diameters (**a**). Dimensionless completeness criterion τ*_full_* (Equation (25)) versus sorbent diameter for three values of initial sorbent concentration (*C_ps_* = 1, 2, and 3 g m^−3^) at fixed contaminant diameter *D_pc_* = 5 µm (**b**). The curves illustrate the model-based theoretical thresholds derived from the comparison of characteristic times.

Figure 7a shows the dependence of the sorbent particle concentration sufficient for contaminant collection on their diameter. The larger the sorbent particles, the higher their concentration is required to be for effective contaminant collection. In the Greenfield gap (shown in the figure by the curve for *D_pc_* = 0.5 µm), the sorbent particle concentration should be higher than for larger contaminant particles.

Figure 7b demonstrates the behavior of the τfull curves (a dimensionless parameter characterizing the completeness of sorption, (25)) depending on the diameter and concentration of sorbent particles. The calculation indicates that, under the specified model assumptions, a sorbent concentration of 2–3 g/m^3^ satisfies the sorption-completeness criterion for contaminant particles up to 10 µm in diameter. At a sorbent concentration of 1 g/m^3^, the criterion is satisfied only for sorbent particles up to approximately 7.5 µm in diameter.

To facilitate practical application of the derived expressions, Table 2 summarizes representative numerical results obtained with the model for selected combinations of sorbent and contaminant diameters and charge conditions. The table lists the approximate overall capture efficiency η, the characteristic capture time *t_capt_*, and the model-based minimum sorbent concentration *C_ps_* _min_.

Thus, within the model, smaller sorbent particles can satisfy the sorption-completeness criterion at lower initial concentrations because their longer airborne residence time provides a greater opportunity for contaminant capture.

The theoretical threshold obtained above is sensitive to processes that are not explicitly represented in the present formulation. Ventilation and air exchange would reduce the airborne residence time of both sorbent and contaminant particles and could therefore increase the sorbent concentration required to satisfy the criterion. Nonuniform contaminant or sorbent distributions would lead to local variations in collision frequency and may result in regions where the local sorbent concentration is below the calculated threshold. Polydispersity would replace the single characteristic particle diameter by a distribution of particle sizes with different settling and capture rates, potentially broadening the range of characteristic times. Particle aggregation could additionally modify the effective particle size, density, surface area, and electrostatic properties, thereby changing both sedimentation and capture rates. Consequently, the calculated *C_ps_* _min_ should be regarded as a model-based theoretical threshold for the specified conditions rather than as a universal concentration required for complete purification of a real indoor space.

## 4. Practical and Toxicological Considerations

The proposed approach involves the intentional introduction of a solid sorbent aerosol into the treated indoor environment. Therefore, the sorbent itself represents a potential secondary exposure that must be considered when evaluating the practical applicability of the method. In the present study, TiO_2_ is used as an example of a solid sorbent, with a characteristic particle diameter of approximately 5–10 µm. The model does not, however, constitute a toxicological assessment of TiO_2_ aerosol exposure and does not establish a safe airborne concentration for human occupancy.

Inhalation exposure to TiO_2_ is relevant because the health effects of inhaled particulate matter depend on particle size, deposited dose, physicochemical properties, and the duration and conditions of exposure. Particle deposition in the respiratory tract is strongly size-dependent: particles larger than approximately 5 µm tend to deposit predominantly in the upper and larger airways, whereas smaller particles can penetrate more deeply into the respiratory system [37,38,39]. Occupational inhalation exposure to TiO_2_ dust has been specifically considered in occupational health assessments, including exposure-control recommendations.

The toxicological evidence for TiO_2_ is dependent on the physicochemical form of the material. In particular, fine and nanoscale TiO_2_ particles should not be treated as toxicologically interchangeable. Inhalation studies and reviews indicate that prolonged exposure to poorly soluble TiO_2_ particles can result in pulmonary particle accumulation and inflammation, while the toxicological response depends on particle size, surface characteristics, crystal form, and deposited dose. The IARC has classified titanium dioxide as possibly carcinogenic to humans (Group 2B) based on the available evidence concerning inhalation exposure; this classification should not, however, be interpreted as a direct estimate of the risk associated with the specific 5–10 µm particles considered in the present model.

Consequently, the calculated sorbent concentrations of approximately 1–2 g/m^3^ should not be interpreted as acceptable concentrations for occupied spaces. They are model-based concentrations required to satisfy the sorption-completeness criterion under the specified aerosol and room assumptions. In particular, the present model does not calculate human inhalation dose, respiratory deposition, occupational exposure limits, or toxicological risk associated with the introduced sorbent aerosol.

For the TiO_2_-based emergency scenario considered here, aerosol deployment should therefore be regarded as an unoccupied-space or evacuated-space operation, unless the safety of a specific sorbent formulation and exposure scenario has been independently demonstrated. Following the treatment period, the sorbent aerosol remaining in the air and deposited on surfaces would need to be removed by appropriate ventilation, filtration, cleaning, or other controlled decontamination procedures before re-entry. The required post-treatment procedure will depend on the sorbent material, particle-size distribution, deposited mass, room ventilation characteristics, and applicable occupational and environmental exposure requirements.

These considerations also limit the practical scope of the present model. The model addresses the physical kinetics of contaminant capture by a dispersed sorbent aerosol; it does not constitute a complete risk assessment of the proposed emergency procedure. Future experimental studies should therefore evaluate not only contaminant-removal efficiency but also sorbent aerosol exposure, respiratory deposition, post-treatment removal, and the conditions required for safe re-occupation of the treated space.

## 5. Conclusions

This paper proposes a mathematical model describing emergency air purification in closed spaces from fine contaminant particles (0.1–5 µm) by a pulsed solid-phase sorbent aerosol, and examines its theoretical behavior through parametric calculations. The novelty of the model lies in the coupling of three previously separate descriptions: (1) the kinetics of pulsed aerosol evolution including gravitational and wall deposition; (2) particle-capture mechanisms (inertial impaction, Brownian diffusion, interception, and electrostatic interaction); and (3) the charge acquired by sorbent particles during pneumatic dispersion.

Under the assumptions and parameter ranges considered in the model, the calculations indicate the following:

1. The contributions of the various capture mechanisms were examined as functions of particle size. Under the assumptions of the model, in the absence of electrical charge the capture efficiency for contaminant particles in the Greenfield gap (0.1–2 µm) does not exceed 0.1–0.3. In this size range, the dominant mechanical mechanism is interception; Brownian diffusion is effective only for particles smaller than ~0.5 µm, while inertial capture remains weak because of the modest difference in size between sorbent and contaminant particles.
2. The calculations indicate that electrostatic charge substantially influences capture efficiency. Under the assumptions and parameter ranges considered, even a charge on the contaminant particles alone significantly increases capture efficiency in the submicron range. When sorbent and contaminant particles carry opposite charges, the calculated capture efficiency in the Greenfield gap rises to 0.7 and higher.
3. Characteristic capture and sedimentation times, together with a criterion for the completeness of sorption, were introduced. Without charging, the capture time in the Greenfield gap is comparable to or longer than the sedimentation time. With oppositely charged particles, the characteristic capture time becomes several times (approximately 3–10) shorter than the sedimentation time, indicating that, under the model assumptions, capture is expected to dominate over sedimentation.
4. An analytical expression was obtained for the model-based minimum sorbent concentration *C_ps_* _min_ corresponding to the sorption-completeness criterion. Under the specified particle properties and model assumptions, the calculations indicate that sorbent particles of 5–7 µm in diameter require approximately 1–2 g m^−3^ to satisfy this criterion, whereas for particles of 10 µm and larger, the calculated threshold increases to approximately 3 g m^−3^ or more. This is explained by the faster gravitational sedimentation of larger particles, which shortens their effective airborne lifetime.
5. The calculations indicate that smaller sorbent particles (≈5 µm rather than ≈10 µm) are preferable under the model assumptions: they remain airborne longer (approximately 29 min versus 8 min), provide higher capture efficiency, and require a lower initial concentration to satisfy the model-based sorption-completeness criterion.

The practical implementation of the proposed approach requires consideration of the sorbent itself as a potential secondary aerosol. For the TiO_2_-based example considered here, the modeled concentrations should not be interpreted as safe exposure levels for occupied spaces. Aerosol deployment should therefore be restricted to unoccupied or evacuated spaces unless the safety of a specific sorbent formulation and exposure scenario has been independently demonstrated. Ventilation, filtration, surface cleaning, and other post-treatment procedures would be required before re-occupation. The present model addresses only contaminant-capture kinetics and does not constitute a toxicological or occupational-exposure assessment.

The practical significance of the work lies in the fact that the obtained relationships allow one to estimate, for specified particle properties and room conditions, the required sorbent size and concentration, the expected capture efficiency, and the characteristic time associated with the modeled reduction in airborne contaminant concentration. The model can therefore support the preliminary design of rapidly deployable systems for emergency response to harmful aerosol releases in industrial, public, and medical facilities.

The concentration thresholds derived in this study should be interpreted strictly within the assumptions of the present formulation. In particular, they do not account for ventilation and air exchange, spatially nonuniform aerosol distributions, particle-size polydispersity, or inter-particle aggregation. These processes may alter residence times, collision frequencies, and effective particle properties, and may therefore change the sorbent concentration required for a given level of contaminant removal. Consequently, the reported values are model-based theoretical indicators rather than operational recommendations.

Future research directions include experimental verification of the model for specific sorbent–contaminant pairs, incorporation of coagulation and aggregation of sorbent particles, and optimization of pulse-spray regimes to minimize sorbent consumption. The present study is limited to theoretical development and parametric evaluation; independent experimental verification remains an essential next step.

## Figures and Tables

**Table 1:** Principal model parameters used in the parametric calculations.

Symbol	Value/Range	Units	Physical Meaning	Source/Status
*H*	5	m	Characteristic room height	Assumed (typical room)
β_0_	0.025	1/m	Empirical wall-deposition coefficient	Experimental ^1^ [23]
*k_D_*_0_	3 × 10^−15^	N·m/K	Empirical diffusion coefficient prefactor	Experimental ^1^ [23]
ρ*_p_* (TiO_2_)	3900–4200	kg/m^3^	Sorbent particle density	Literature
ρ*_p_* (carbon/coal dust)	1500–2000	kg/m^3^	Contaminant particle density (example)	Literature/assumed
*D_ps_*	5–10	µm	Sauter-mean sorbent diameter	Parametric range
*D_pc_*	0.1–5	µm	Sauter-mean contaminant diameter	Parametric range (Greenfield gap focus)
*C_ps_*(0)	1–3	g/m^3^	Initial sorbent mass concentration	Parametric range
*C_pc_*(0)	1	g/m^3^	Initial contaminant mass concentration (example)	Assumed for illustrations
*q*_0_ (contaminant)	2	µC/m^2^	Surface charge ^1^ density coefficient (bacteria/viruses)	Estimated from [30,31]
*q_ps_*	10^−12^ D_ps_^2^	C	Charge ^1^ of sorbent particle (opposite sign)	Estimated from triboelectric data [24,32,33]
ε (TiO_2_)	≈80–100	–	Relative permittivity of sorbent	Literature
*T*	293	K	Absolute temperature	Standard conditions
μ	1.8 × 10^−5^	Pa·s	Dynamic viscosity of air	Standard conditions
λ	≈68	nm	Mean free path of air molecules	Standard conditions
*g*	9.81	m/s^2^	Gravitational acceleration	Physical constant

^1^ Parameters marked “Experimental” were obtained for pulsed titanium-oxide aerosols in earlier work. Charge-related quantities are order-of-magnitude estimates; their influence is examined parametrically. Plausible ranges for charge and density should be refined for any specific material pair.

**Table 2:** Representative numerical examples obtained with the model equations.

Case	*D_ps_*, μm	*D_pc_*, μm	Charge Condition	η	*t_capt_* (min)	*C_psmin_*, g m^−3^
1	5	0.5	Uncharged	0.5–0.25	Long	>3
2	5	0.5	Opposite charges	>0.7	<<*t_dept_*	~1–2
3	10	0.5	Opposite charges	~0.5–0.7	Intermediate	~2–3
4	5	5	Uncharged/opposite charges	~1	Short	~1
5	10	5	Opposite charges	~1	Short	~2

## Data Availability

The data presented in this study are available upon request from the corresponding author.

## Nomenclature

*C*	Mass concentration of aerosol, g/m^3^
*C_pc_*	Mass concentration of contaminant particles, g/m^3^
*C_ps_*	Mass concentration of sorbent particles, g/m^3^
*C_ps_* _min_	Model-based minimum sorbent concentration satisfying the sorption-completeness criterion, g/m^3^
*D_pc_*	Diameter of contaminant particle (Sauter mean diameter), m, µm
*D_ps_*	Diameter of sorbent particle (Sauter mean diameter), m, µm
*D*_min_	Particle diameter corresponding to minimum sedimentation rate, m, µm
*E* *_electr_*	Electric field strength, V/m
*g*	Gravitational acceleration (9.81), m/s^2^
*H*	Characteristic dimension of the room (height), m
*k_B_*	Boltzmann constant (1.38 × 10^−23^), J/K
*k_D_*	Diffusion coefficient of a particle, m^2^/s
*k_D_*_0_	Empirical parameter in diffusion coefficient (3 × 10^−15^), N·m/K
*m_p_*	Mass of settled particles, g
*m_pc_*	Mass of settled contaminant particles, g
*q*	Electric charge of a particle, C
*q_pc_*	Electric charge of contaminant particle, C
*q_ps_*	Electric charge of sorbent particle, C
*q*_0_	Surface charge density coefficient (2, µC/m^2^)
*r*	Distance between particle centers, m
*R*	Gas constant, J/(mol·K)
*t*	Time, s
*t_capt_*	Characteristic capture time (1/(K⋅C_ps_)), s
*t_dept_*	Characteristic sedimentation time (H/u_s_), s
*T*	Absolute temperature, K
*u_s_*	Stokes settling velocity, m/s
*U*	Relative velocity between PSs and PCs, m/s
β	Wall deposition coefficient, 1/m
β_0_	Empirical parameter in wall deposition (0.025), 1/m
γ	Overall sedimentation rate constant, 1/s
ε_0_	Vacuum permittivity (8.85 × 10^−12^), F/m
ε	Relative dielectric permittivity of particle
η	Overall capture efficiency
η_dif_	Capture efficiency due to Brownian diffusion
η_elec_	Capture efficiency due to electrostatic interaction
η_iner_	Capture efficiency due to inertial impaction
η_inter_	Capture efficiency due to interception
λ	Mean free path of air molecules (≈68), nm
μ	Dynamic viscosity of air, Pa·s
ρ_p_	Particle density, kg/m^3^
τ_full_	Dimensionless criterion for complete sorption
Dimensionless numbers	
Kn	Knudsen number (λ/(*D_p_*/2))
N_E_	Electrostatic interaction parameter
Pe	Péclet number (Equation (11))
Stk	Stokes number (Equation (9))
Subscripts	
c	Contaminant (PC—contaminant particle)
s	Sorbent (PS—sorbent particle)
*p*	Particle (general)
0	Initial condition (*t* = 0)
